# Immunoprofiling Reveals Novel Mast Cell Receptors and the Continuous Nature of Human Lung Mast Cell Heterogeneity

**DOI:** 10.3389/fimmu.2021.804812

**Published:** 2022-01-04

**Authors:** Elin Rönnberg, Daryl Zhong Hao Boey, Avinash Ravindran, Jesper Säfholm, Ann-Charlotte Orre, Mamdoh Al-Ameri, Mikael Adner, Sven-Erik Dahlén, Joakim S. Dahlin, Gunnar Nilsson

**Affiliations:** ^1^ Division of Immunology and Allergy, Department of Medicine Solna, Karolinska Institutet, and Karolinska University Hospital, Clinical Immunology and Transfusion Medicine, Stockholm, Sweden; ^2^ Centre for Allergy Research, Karolinska Institutet, Stockholm, Sweden; ^3^ Unit for Experimental Asthma and Allergy Research, The Institute of Environmental Medicine, Karolinska Institutet, Stockholm, Sweden; ^4^ Thoracic Surgery, Department of Molecular Medicine and Surgery, Karolinska Institutet, and Karolinska University Hospital, Stockholm, Sweden; ^5^ Department of Medical Sciences, Uppsala University, Uppsala, Sweden

**Keywords:** human lung mast cells, heterogenity, chymase (CMA1), carboxypeptidase A3 (CPA3), SUSD2, CD38, FcεRI

## Abstract

**Background:**

Immunohistochemical analysis of granule-associated proteases has revealed that human lung mast cells constitute a heterogeneous population of cells, with distinct subpopulations identified. However, a systematic and comprehensive analysis of cell-surface markers to study human lung mast cell heterogeneity has yet to be performed.

**Methods:**

Human lung mast cells were obtained from lung lobectomies, and the expression of 332 cell-surface markers was analyzed using flow cytometry and the LEGENDScreen™ kit. Markers that exhibited high variance were selected for additional analyses to reveal whether they were correlated and whether discrete mast cell subpopulations were discernable.

**Results:**

We identified the expression of 102 surface markers on human lung mast cells, 23 previously not described on mast cells, of which several showed high continuous variation in their expression. Six of these markers were correlated: SUSD2, CD49a, CD326, CD34, CD66 and HLA-DR. The expression of these markers was also correlated with the size and granularity of mast cells. However, no marker produced an expression profile consistent with a bi- or multimodal distribution.

**Conclusions:**

LEGENDScreen analysis identified more than 100 cell-surface markers on mast cells, including 23 that, to the best of our knowledge, have not been previously described on human mast cells. The comprehensive expression profiling of the 332 surface markers did not identify distinct mast cell subpopulations. Instead, we demonstrate the continuous nature of human lung mast cell heterogeneity.

## Introduction

Heterogeneity among mast cells has been known for a long time and was first attributed to differential expression of proteoglycans in rodent mast cells, which gave them distinct staining patterns (1). This led to the division of rodent mast cells into connective tissue mast cells and mucosal mast cells. In humans, mast cell heterogeneity has been based on the expression of mast cell proteases, i.e., cells expressing tryptase only (MC_T_) and those expressing both tryptase and chymase (MC_TC_) as well as carboxypeptidase A (2, 3). These subtypes have been defined using immunohistochemistry, a method that produced binary results, that is, the absence or presence of expression. The MC_TC_ subtype is more predominant in connective tissues such as the skin, while the MC_T_ subset is more prevalent in mucosal surfaces such as the airways and gastrointestinal tract (4).

Mast cells are found in the human lungs in all different compartments, i.e., under the epithelium, in smooth muscle bundles, around pulmonary vessels, in the parenchyma and in close proximity to sensory nerves (5). Human lung mast cells (HLMCs) have several important functions in health and diseases, such as host defense, induction of acute inflammatory responses, vascular regulation, bronchoconstriction and tissue remodeling (6–9). The heterogeneity of HLMCs was first suggested to be related to differences in their size and function (10), where a heterogeneity in the response to secretagogues also was reported (11). It was described that the protease expression within HLMCs differ, with the MC_T_ being the predominant subtype except around pulmonary vessels, where the MC_T_ and MC_TC_ subtypes are found in equal numbers (2). However, the heterogeneity among HLMCs goes beyond size and protease expression, as demonstrated by the differential expression of certain mast cell-related markers (FcεRI, IL-9R, 5-LO, LTC_4_S, etc.) among the MC_T_ and MC_TC_ populations in different lung compartments (12).

Mast cell heterogeneity has primarily been studied in a binary manner using immunohistochemistry, describing the absence or presence of a given marker. Here, we used a quantitative flow cytometry-based approach to study HLMC heterogeneity, profiling the expression of 332 surface markers and intracellular staining of the proteases tryptase, chymase and CPA3. None of these markers distinctly divided the HLMCs studied into subpopulations. However, several markers showed a high degree of variation within the mast cell population with a nonclustered gradient expression pattern. Six of these markers correlated with each other, revealing the continuous nature of HLMC heterogeneity rather than separation into distinct subpopulations.

## Materials and Methods

### Ethical Approval

The local ethics committee approved the collection of lung tissue from patients undergoing lobectomy, and all patients provided informed consent (Regionala Etikprövningsnämnden Stockholm, 2010/181-31/2).

### Cell Preparation

Single-cell suspensions were obtained from macroscopically healthy human lung tissue as previously described (13). Briefly, human lung tissue was cut into small pieces and enzymatically digested for 45 min with DNase I and collagenase. Thereafter, the tissue was mechanically disrupted by plunging through a syringe, the cells were washed, and debris was removed by 30% Percoll centrifugation. After preparation, the cells were stained and analyzed by flow cytometry.

### Flow Cytometry

The following antibodies were used: anti-CD45-V500 (Clone HI30, BD Biosciences, San Jose, CA, USA), anti-CD14-APC-Cy7 (clone M5E2, BioLegend, San Diego, CA, USA), anti-CD117-APC (clone 104D2, BD Biosciences), anti-FcεRI-FITC (clone CRA1, Miltenyi Biotec, Bergisch Gladbach, Germany), anti-FcεRI-PE (clone CRA1, BioLegend), anti-SUSD2-PE (clone W3D5, BioLegend), anti-CD63- FITC (clone H5C6, BD Biosciences), anti-CD49a-BV786 (clone SR84, BD Biosciences), anti-CD66a/c/e-A488 (clone ASL-32, BioLegend), anti-CD326-BV650 (clone 9C4, BioLegend), anti-CD34-BV421 (clone 581, BD Biosciences), anti-HLA-DR-PE/Cy5 (clone L243, BioLegend), anti-CD344-PE/Vio770 (clone CH3A4A7, Miltenyi Biotec), anti-CD38-BV421 (clone HIT2, BD Biosciences), anti-tryptase (clone G3, Millipore, Burlington, MA, USA) conjugated in-house with an Alexa Fluor 647 monoclonal antibody labeling kit (Thermo Fisher Scientific, Waltham, MA, USA), anti-CPA3 antibodies (clone CA5, a kind gift from Andrew Walls, Southampton, UK) conjugated in-house with an Alexa Fluor™ 488 antibody labeling kit (Thermo Fisher Scientific) and chymase (clone B7, Millipore) conjugated in-house with a PE Conjugation Kit (Abcam, Cambridge, UK). Surface staining was performed by incubation for 30 min at 4 degrees with the antibodies in PBS+ 2% FBS, followed by washing with PBS+ 2% FBS. When using the LEGENDScreen™ human cell screening kit, which contains 332 markers and 10 isotype controls, conjugated to PE (Cat. 70001, BioLegend) that are detailed in **Supplementary Table S1**, cells were first stained with anti-CD45, anti-CD117, anti-CD14 and anti-FcεRI antibodies for 30 min, followed by washing. Thereafter, the cells were stained with the kit reagents according to the manufacturer’s instructions. The cells from each donor were not sufficient to run an entire screen. Therefore, each kit was run using several donors, and a total of 10 donors were used to run three Legendscreens kits. The presence of anti- FcεRI antibody in the backbone panel did not result in mast cell activation, as measured by surface CD63 expression (data not shown). For intracellular staining, cells were fixed with 4% paraformaldehyde (PFA) and permeabilized using 0.1% saponin in PBS with 0.01 M HEPES (PBS-S buffer). Nonspecific binding was blocked using blocking buffer (PBS-S with 5% dry milk and 2% fetal calf serum (FCS)). The cells were thereafter stained intracellularly over-night with the antibodies in blocking buffer, thereafter washed with PBS-S buffer and PBS+ 2% FBS. The cells were analyzed using a BD FACSCanto (BD, Franklin Lakes, NJ, USA) or BD LSRFortessa, and FlowJo software version 10 (FlowJo LLC, Ashland, OR, USA) was used for flow cytometry data analysis. Fluorescent minus one (FMO) controls (extracellular) or isotype controls (intracellular) were used to establish gates for positive staining (**Supplementary Figure S1**).

### Statistical Analysis

**Figure 1:**
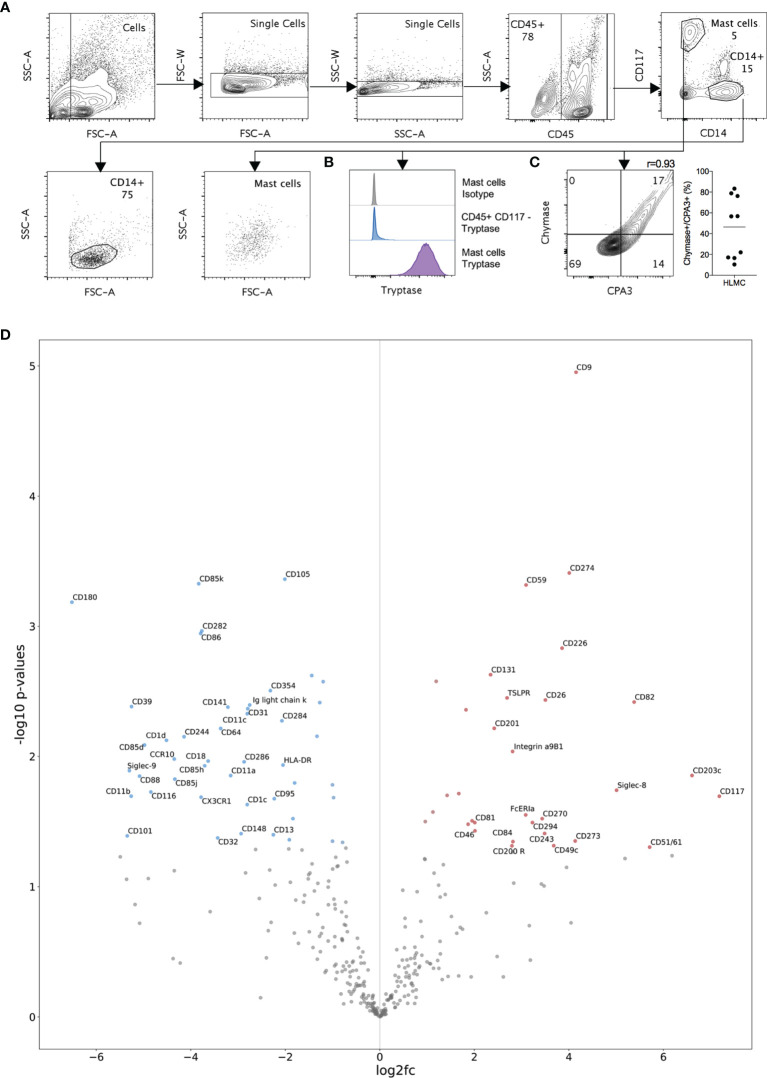
Protease expression and LEGENDScreen analysis of HLMC. **(A)** Representative gating strategies for HLMCs and CD14^+^ cells are shown. **(B)** Intracellular tryptase-, **(C)** chymase- and CPA3- stained HLMCs and quantification of CPA3^+^/Chymase^+^ cells n = 9. **(D)** Comparison of marker expression included in the LEGENDScreen kit between mast cells and CD14^+^ cells. Volcano plot showing log2-fold change in mast cells divided by CD14^+^ cells (normalized MFI values with the plate-matched FMO subtracted) against -log10 p-values (independent 2-sided t-test) of mast cells against CD14^+^ cells. Markers are annotated only if abs(log2fc) => 2 and p-value < 0.05. n = 3.

**Figure 2:**
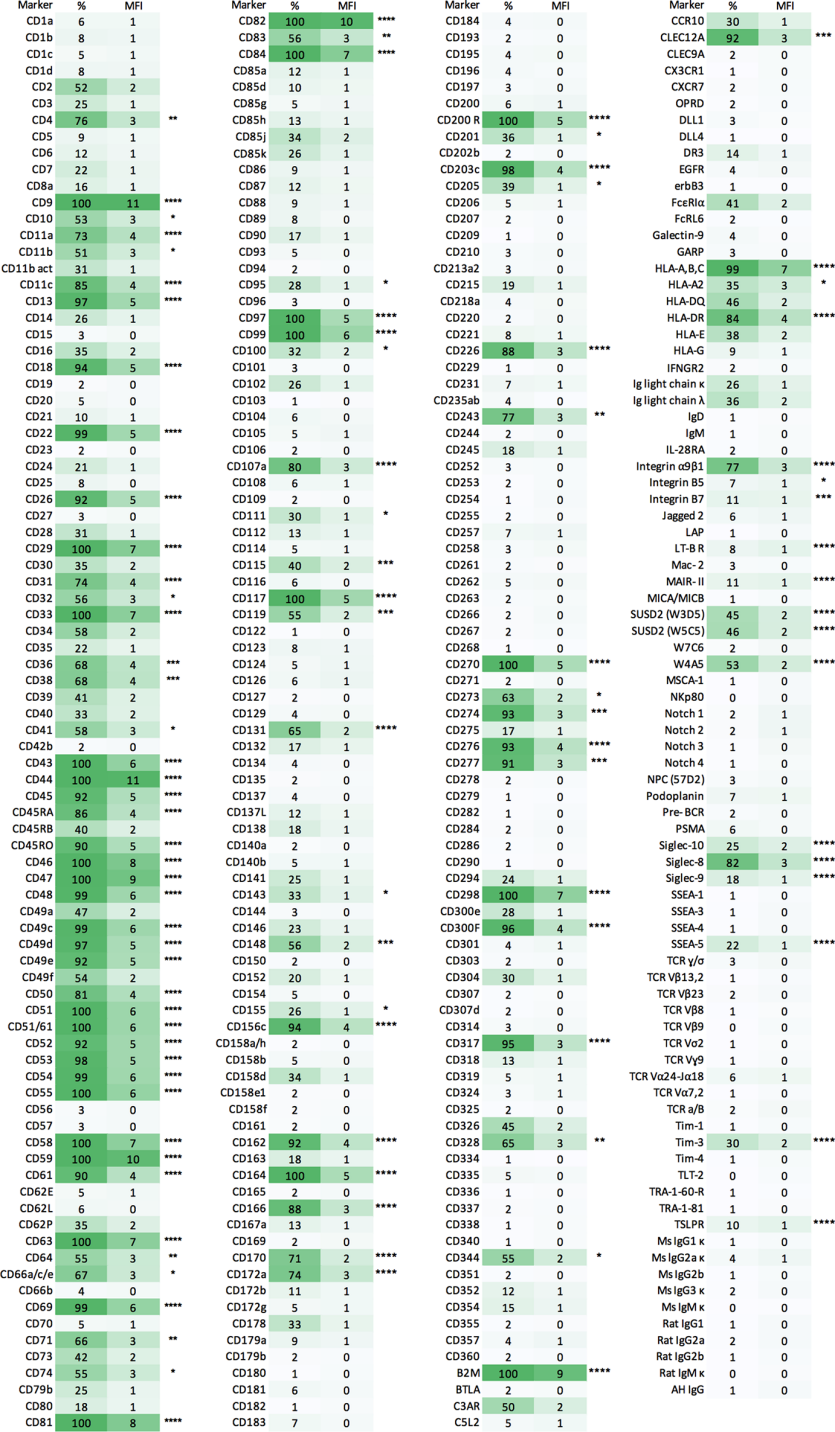
Expression of cell-surface antigens on HLMCs. HLMCs stained with the LEGENDScreen human cell screening kit. Mast cells were gated as CD45^+^CD14^low^CD117^high^ cells. Shown are the percent positive (%) for each marker, the MFI (normalized to the plate-matched FMO control and log_10_ transformed) and the stars represents the significance of the MFI of the marker compared to that of the FMO control (one-way ANOVA with Dunnett’s multiple comparisons test). *p < 0.05, **p < 0.01, ***p < 0.001, ****p < 0.0001. n = 3.

Statistical analyses were performed with GraphPad Prism software version 7.0b (**Figures 1C**, **2** and **4**) or the Python environment (3.7) with the following packages: statsmodels (0.10.1), seaborn (0.9.0), scipy (1.4.1), pandas (1.1.0), numpy (1.18.1), and matplotlib (3.1.3) (**Figure 1D**). The specific methods used are detailed in the figure legends. * p < 0.05; ** p < 0.01; *** p<0.001; **** p<0.0001.

## Results

### Expression of Mast Cell Proteases

The classic division into distinct human mast cell subpopulations is based on the presence or absence of granule-associated antigens, i.e., the proteases tryptase, chymase and CPA3. We therefore first performed intracellular staining of lung cells and used a flow cytometry-based readout to accurately characterize the mast cell population with respect to the expression of tryptase, chymase and CPA3. Flow cytometry analysis identified CD45^+^CD14^low^CD117^high^ HLMCs with the characteristic expression of tryptase (**Figures 1A, B**). The HLMC population showed a high variation of chymase and CPA3 expression between individual cells and the degree of CPA3 and chymase double positive cells varied considerably from donor to donor (**Figure 1C**). However, no distinct subpopulations were discernable, instead there was a continuous spectrum of expression levels.

### Immunoprofiling of HLMCs

Whereas intracellular antigens failed to discriminate distinct subpopulations, we set out to perform an extensive mapping of cell surface antigens on HLMCs to potentially identify distinct subpopulations and novel markers. Flow cytometry analysis characterized the expression of 332 surface markers on HLMCs, using a LEGENDScreen™ human cell screening kit. Since many of the markers are broadly expressed we used CD14^+^ cells as a reference to selectively enrich for lineage-specific antigens in HLMCs (**Figure 1D**). Well-known monocyte markers such as CD11b, CD11c, and HLA-DR were highly expressed on CD14^+^ cells, verifying the validity in enriching for lineage-characteristic markers. In analogy, HLMCs expressed high levels of CD117 and FcεRI. Markers with the most significant differences between the HLMCs and CD14^+^ cells included CD9, CD59, CD274 and CD226 (**Figure 1D**). CD9 is a broadly expressed tetraspanin with a wide variety of functions; in mast cells, it is abundantly expressed and has been implicated in chemotaxis and activation (14). CD59 can prevent complement-induced cytolytic cell death by preventing assembly of the complement membrane attack complex and has also been implicated in T cell activation (15). CD274 is also known as programmed death ligand-1 (PD-L1) and can cause blockade of T cell activation (16). CD226 has received increasing interest in recent years and can play a role in many immunological processes (17), including enhancement of FcεRI-mediated activation in mast cells (18).

Of the 332 markers analyzed, HLMCs showed significant expression of 102 markers (**Figure 2**), of which 23, to the best of our knowledge, have not been described on (non-neoplastic) human MCs before (**Table 1**).

**Table 1:** Novel antigens identified on HLMCs.

Marker (clone)	Description
**CD36**	Receptor binding a broad range of lipids
**CD45RO**	Isoform of CD45
**CD66a/c/e**	Adhesion molecules
**CD74**	Involved in MHC class II antigen processing and a receptor for macrophage migration inhibitory factor
**CD111**	Adhesion molecule
**CD115**	Receptor for M-CSF and IL-34
**CD131**	Common β subunit of the IL-3, IL-5 and GM-CSF receptors
**CD143**	Metallopeptidase
**CD148**	Tyrosine phosphatase involved in signal transduction
**CD164**	Sialomucin involved in cell adhesion and proliferation
**CD166**	Glycoprotein involved in cell adhesion and migration
**CD205**	Endocytic receptor involved in antigen uptake and processing
**CD243**	Involved in transportation of molecules across cell membranes
**CD270**	Receptor for TNFSF14, BTLA, LTA and CD160
**CD277**	Regulate T cell responses
**CD317**	Blocks the release of certain viruses from infected cells
**CD344 (Frizzled-4)**	Receptor for Wnt proteins and norrin
**CLEC12A/CD371**	C-type lectin-like receptor with an immunoreceptor tyrosine-based inhibitory motif (ITIM) domain
**Integrin α9β1**	Integrin mediating cell adhesion and migration
**SUSD2 (W3D5, W5C5)**	Potentially involved in cell adhesion as this transmembrane protein contains functional domains associated with adhesion molecules
**(W4A5)**	Antigen has yet to be described
**Siglec-9**	Lectin that binds sialic acid and has ITIM domains
**SSEA-5**	A glycan

### Heterogeneous Expression of the High-Affinity IgE Receptor FcεRI

**Figure 3:**
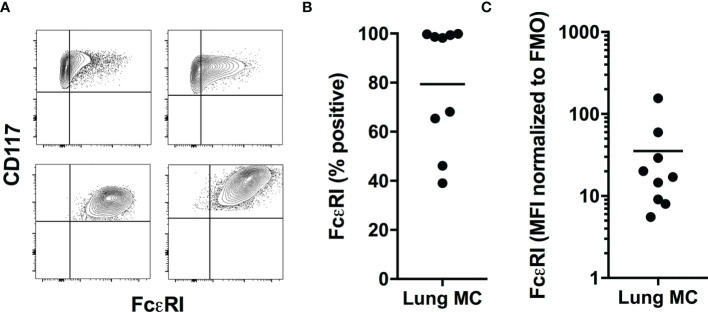
FcεRI expression on HLMCs. Examples of CD117/FcεRI expression on HLMCs gated as CD45^+^CD14^low^CD117^high^ cells from four donors **(A)**. Quantification of the percent positive for FcεRI **(B)** and the MFI of FcεRI normalized to that of the matched FMO control **(C)**. n = 9.

One of the principal markers for MCs is the expression of the high-affinity IgE-receptor FcεRI. FcεRI expression on HLMCs has been shown to differ among compartments in the lungs, as well as between healthy and diseased tissues. HLMCs from healthy individuals present in the parenchyma are negative for FcεRI, while patients with concurrent asthma show higher expression of the receptor (12, 19). In our study, the expression varied considerably between donors and there was no clear separation between the negative and positive FcεRI populations but rather a continuous spectrum of expression and approximately 50% of the donors expressed FcεRI on virtually all MCs (**Figures 3A**–**C**).

### Heterogeneous Expression of Cell-Surface Markers With a Continuous Distribution

**Figure 4:**
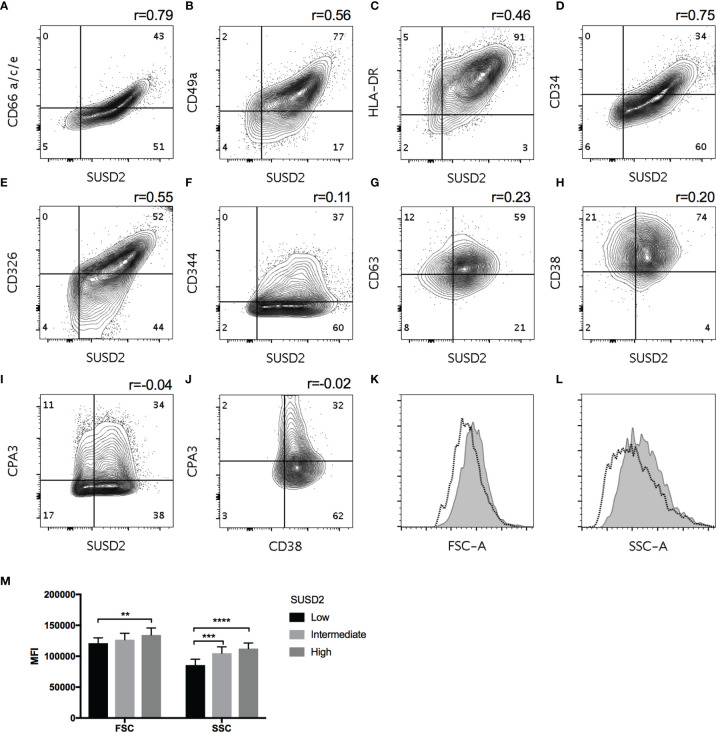
Co-staining of HLMCs with indicated markers. Pearson correlations in each donor was calculated and the average r is shown (n = 3-4) **(A–J)**. SUSD2low, intermediate and high cells were gated, and the FSC **(K)** and SSC **(L)** values of the SUSD2low (dotted line) and SUSD2high (filled gray) populations are shown. Quantification of FSC and SSC is shown in **(M)**, mean ± SEM, n = 5. Two-way ANOVA with Bonferroni’s multiple comparisons test was performed. **p < 0.01; ***p < 0.001; ****p < 0.0001.

The heterogeneity of HLMCs has primarily been studied using immunohistochemistry in a binary manner and they have been divided into the MC_T_ and MC_TC_ subtypes (2, 3). How this heterogeneity is reflected by the heterogeneous expression of cell-surface markers has barely been investigated. None of the surface markers investigated in this study, using quantitative flow cytometry, did distinctly and consistently divide the HLMCs into subpopulations (data not shown). However, several markers were expressed with a considerable continuous variation within the HLMC population (**Table 2**). Co- stainings of nine of these markers revealed that expression of six of the markers, SUSD2, CD49a, CD326, CD34, CD66 and HLA-DR correlated (r > 0. 4) (**Figures 4A**–**H**). The correlations seen between markers was not due to autofluorescence since there was no correlations in the FMO controls (**Supplementary Figures S1A**–**E**). In addition, the HLMCs showed no significant staining with the 10 isotype controls (**Figure 2**).

**Table 2:** The 10 markers from the LEGENDScreen analysis with the highest robust coefficient of variation (robust CV).

Marker	Robust CV
**SUSD2 (W5C5)**	264
**SUSD2 (W3D5)**	246
**CD344**	172
**CD49a**	160
**CD326**	155
**CD66a/c/e**	153
**CD34**	134
**HLA-DR**	133
**SSEA-5**	131
**CD63**	124
**CD38**	123

We next investigated if the expression of these markers correlated to the classical mast cell subsets using intracellular staining for CPA3 as a marker for MC_TC_. Expression of neither SUSD2, CD38 (**Figures 4I, J**) nor CD344 (data not shown) correlated with CPA3 expression.

Since the expression of SUSD2, a marker for stem/progenitor cells (20), correlated to CD34, that is expressed on circulating mast cell progenitors (21), we investigated if the expression of these markers could identify MCs in different stages of maturation. When mast cells mature they become larger with an increasing number of granules (22). We therefore gated SUSD2 low, intermediate and high cells and compared the FSC and SSC values that reflect the size and granularity of the cells. However, SUSD2high cells had higher FSC and SSC then SUSD2low cells (**Figures 4K**–**M**), suggesting that they were larger with a higher granularity and therefore unlikely to be immature MCs.

SUSD2 has been linked to proliferation in cancer cells (23), why we investigated the proliferation status of the cells with the proliferation marker Ki-67. However, in agreement with the fact that mast cells are long-lived cells with low turnover (24), no staining was observed (**Supplementary Figure S2**).

## Discussion

HLMCs have been shown to be heterogeneous; classically, they have been studied using immunohistochemistry in a binary manner, and they have been divided into the MC_T_ and MC_TC_ subtypes based on whether the mast cell proteases chymase and CPA3 are detectable (2, 3). By using a quantitative flow cytometry based method we indeed found high variation in chymase and CPA3 expression but no distinct subpopulations were discernable (**Figure 1C**). The apparent discrepancy between our study and previous immunohistochemical studies is probably due to the fact that we have studied the expression in a quantitative manner using flow cytometry, thus finding that there is a spectrum of different expression levels. In contrast, in previous studies the cells have been classified into MC_T_/MC_TC_ cells in a binary manner depending on the detection limit of the immunohistochemical technique.

Although attempts have been made to map cell-surface antigens on HLMCs (25–30), extensive mapping including the heterogeneity of cell-surface antigen expression has not been carried out. In this study, we identified significant expression of 102 markers on the HLMC surface (**Figure 2**), of which, to the best of our knowledge, 23 are novel mast cell markers (**Table 1**). Several of these markers, including SSEA-5, SUSD2, W4A5, CD243, CD111, CD131 and CD164, are described as markers expressed on stem cells. The expression of stem cell markers on mast cells is in accordance with results from the FANTOM5 consortium, in which skin mast cells exhibited similarities with stem cells (31). In some cases, our results are in disagreement with previously published data; for example, CD4, CD10, CD36 and CD74 were previously shown to not be expressed by HLMCs (26, 27). This discrepancy might be explained by differences in the procedures, as in contrast to published data, we did not purify or culture the studied mast cells prior to analysis (25, 27, 29, 30). Culturing mast cells has been shown to alter their phenotype and expression of cell-surface receptors (31, 32). Furthermore, although macroscopically healthy tissue distal from the tumor was used for our analysis, one cannot rule out the possibility that also this part of the tissue is affected by the disease and can have an impact on the results.

The expression of FcεRI has been shown to differ in different compartments of the lung, with mast cells present in the parenchyma being negative for FcεRI (12). We have investigated single cell suspensions from lung tissues without separation of the parenchymal cells. However, we did not observe distinct FcεRI positive and negative mast cell populations but rather a continuous spectrum of expression levels (**Figure 3A**). Additionally, in about 50% of the donors virtually all mast cells stained positive for FcεRI, meaning that we cannot detect any FcεRI negative parenchymal mast cell population in these individuals (**Figure 3A**). This discrepancy is again likely to be due to the different techniques used, immunohistochemistry and flow cytometry, and the different detection limits of the techniques. We also observed a large variation in expression of FcεRI among individuals (**Figures 3A**–**C**), and in line with our results, this has previously been shown also for human skin mast cells (33). The reasons for this variation could be manifold, as the surface expression of FcεRI can be regulated in many different ways. FcεRI is, for example, upregulated by IL-4 and stabilized on the cell surface by the binding of IgE antibodies (34). Furthermore, it was described recently that IL-33 downregulates the expression of FcεRI (35, 36), indicating that the state of inflammation in the tissue can influence FcεRI expression.

Heterogeneous expression of cell-surface markers on mast cells has scarcely been investigated. We investigated the heterogeneity of cell-surface markers in a quantitative manner using flow cytometry and did not find any markers that distinctly and consistently divided the studied mast cells into subpopulations with a bi- or multimodal distribution (data not shown). We did, however, find several markers with considerable continuous variation in expression within the mast cell populations (**Table 2**), and co-stainings revealed that six of these markers, SUSD2, CD49a, CD326, CD34, CD66 and HLA-DR, were correlated (**Figure 4**). To investigate whether these markers are correlated with the classic mast cell subpopulations MC_T_ and MC_TC_, we costained for SUSD2 and CPA3, but no correlation was detected, ruling out the possibility that these markers are extracellular markers of the classic mast cell subtypes (**Figure 4I**). Recently, CD38 was demonstrated to be differentially expressed in human nasal polyp mast cells where CD38^low^ were of the MC_TC_ subtype, while CD38^high^ MCs were a heterogenous pool of both MC_T_ and MC_TC_ cells (37). In our study, CD38 did not distinctly separate the HLMCs into subpopulations and did not correlate with CPA3; i.e., in HLMCs CD38 could not be used to distinguish the MC_T_ and MC_TC_ subtypes (**Figure 4J**). CD344 did not correlate with the MC_T_ or MC_TC_ profile either (data not shown). Another marker suggested to be expressed on MC_TC_ is the complement 5a receptor CD88 (38). However, we did not detect significant expression of CD88 on HLMCs (**Figure 2**). Thus, we were unable to find an extracellular marker that distinguishes the classical mast cell subsets.

One of the markers that showed high continuous variation in the HLMCs, CD63, is used as a surrogate marker for mast cell activation (39), i.e., degranulation. However, CD63 did not correlate to any of the other eight markers investigated, including the 6 markers that show correlation to each other SUSD2, CD49a, CD326, CD34, CD66 or HLA-DR indicating that these markers do not reflect varying degree of activation (**Figure 4G** and data not shown).

Since two of our six heterogeneity markers that correlate, CD34 and SUSD2, is expressed on stem/progenitor cells (20, 21, 40), we wondered whether these markers could identify cells in different stages of mast cell maturation. However, if that was the case, one would expect cells with high expression of SUSD2/CD34 to be small and contain few granules similar to mast cell progenitors (21). In contrast, the cells with high expression of SUSD2 had relatively high FSC and SSC values (**Figures 4K**–**M**), suggesting that they were relatively large and granular and therefore unlikely to be immature mast cells. In this context, it is worth noting that mature murine mast cells also express CD34 (41), and in these cells, CD34 inhibits adhesion and is required for optimal migration (42).

SUSD2 is also expressed in certain cancers, in which it has been linked to proliferation (23); thus, one could imagine that cells with high SUSD2 expression are proliferating. However, we could not detect any staining for the proliferation marker Ki67 in HLMCs (**Supplementary Figure S2**).

In summary, we have identified the expression of 102 cell-surface antigens on HLMCs, of which 23 have not been described previously on MCs. Several of these antigens had a high continuous variability in their expression within the HLMC population. The expression of six of these markers correlated to each other and the size and granularity of the cells. Further studies are needed to determine how these cells differ functionally. None of the markers correlated with the intracellular protease expression. Thus, in contrast to the dogma of distinct mast cell subtypes, we demonstrate the continuous nature of HLMC heterogeneity.

## Data Availability Statement

The original contributions presented in the study are included in the article/**Supplementary Material**. Further inquiries can be directed to the corresponding authors.

## Ethics Statement

The studies involving human participants were reviewed and approved by Regionala Etikprövningsnämden, Stockholm. The patients/participants provided their written informed consent to participate in this study.

## Author Contributions

ER, JD, and GN conceived and designed the studies. ER, JD, and AR designed and performed the experiments. ER, DZ, and JD analyzed the data. JS, A-CO, MA-A, MA, and S-ED provided samples. ER, GN, and JD wrote the manuscript draft. All authors reviewed, critically revised, and approved the final manuscript.

## Funding

This study was supported by grants from the Swedish Research Council (2018-02070 and 2020-01693); the Heart-Lung Foundation; The Swedish Cancer Society; the Ellen, Walter and Lennart Hesselman Foundation; the Tore Nilsson Foundation; the Lars Hierta Memorial Foundation; the Konsul Th C Bergh Foundation; the Tornspiran Foundation; the O. E. and Edla Johanssons Foundation; the Swedish Society for Medical Research; The Centre for Allergy Research Highlights Asthma Markers of Phenotype (ChAMP) consortium funded by the Swedish Foundation for Strategic Research; the AstraZeneca & Science for Life Laboratory Joint Research Collaboration; and the Karolinska Institutet. The funding sources were not involved in the study design, collection and interpretation of data, writing the report or the decision to submit the article for publication.

## Conflict of Interest

S-ED reports personal fees from AstraZeneca, Cayman Chemicals, GSK, Novartis, Regeneron, Sanofi, and Teva, for consultancies outside the submitted work.

The remaining authors declare that the research was conducted in the absence of any commercial or financial relationships that could be construed as a potential conflict of interest.

## Publisher’s Note

All claims expressed in this article are solely those of the authors and do not necessarily represent those of their affiliated organizations, or those of the publisher, the editors and the reviewers. Any product that may be evaluated in this article, or claim that may be made by its manufacturer, is not guaranteed or endorsed by the publisher.

## References

[1] Enerback L. Mast Cells in Rat Gastrointestinal Mucosa. 2. Dye-Binding and Metachromatic Properties. Acta Pathol Microbiol Scand (1966) 66:303–12. doi: 10.1111/apm.1966.66.3.303 4162018

[2] Irani AA Schechter NM Craig SS Deblois G Schwartz LB. Two Types of Human Mast Cells That Have Distinct Neutral Protease Compositions. Proc Natl Acad Sci USA (1986) 83:4464–8. doi: 10.1073/pnas.83.12.4464. PMC323754 3520574

[3] Irani AM Goldstein SM Wintroub BU Bradford T Schwartz LB. Human Mast Cell Carboxypeptidase. Selective Localization to MCTC Cells. J Immunol (1991) 147:247–53. 2051021

[4] Metcalfe DD Baram D Mekori YA. Mast Cells. Physiol Rev (1997) 77:1033–79. doi: 10.1152/physrev.1997.77.4.1033 9354811

[5] Balzar S Fajt ML Comhair SA Erzurum SC Bleecker E Busse WW. Mast Cell Phenotype, Location, and Activation in Severe Asthma. Data From the Severe Asthma Research Program. Am J Respir Crit Care Med (2011) 183:299–309. doi: 10.1164/rccm.201002-0295OC 20813890 PMC3056228

[6] Holgate ST Hardy C Robinson C Agius RM Howarth PH. The Mast Cell as a Primary Effector Cell in the Pathogenesis of Asthma. J Allergy Clin Immunol (1986) 77:274–82. doi: 10.1016/S0091-6749(86)80104-X 2418090

[7] Bradding P Walls AF Holgate ST. The Role of the Mast Cell in the Pathophysiology of Asthma. J Allergy Clin Immunol (2006) 117:1277–84. doi: 10.1016/j.jaci.2006.02.039 16750987

[8] Erjefalt JS. Mast Cells in Human Airways: The Culprit? Eur Respir Rev (2014) 23:299–307. doi: 10.1183/09059180.00005014 25176966 PMC9487311

[9] Arthur G Bradding P. New Developments in Mast Cell Biology: Clinical Implications. Chest (2016) 150:680–93. doi: 10.1016/j.chest.2016.06.009 27316557

[10] Schulman ES Kagey-Sobotka A Macglashan DW Jr Adkinson NF Jr Peters SP Schleimer RP. Heterogeneity of Human Mast Cells. J Immunol (1983) 131:1936–41. 6194221

[11] Lowman MA Rees PH Benyon RC Church MK. Human Mast Cell Heterogeneity: Histamine Release From Mast Cells Dispersed From Skin, Lung, Adenoids, Tonsils, and Colon in Response to IgE-Dependent and Nonimmunologic Stimuli. J Allergy Clin Immunol (1988) 81:590–7. doi: 10.1016/0091-6749(88)90199-6 2450114

[12] Andersson CK Mori M Bjermer L Lofdahl CG Erjefalt JS. Novel Site-Specific Mast Cell Subpopulations in the Human Lung. Thorax (2009) 64:297–305. doi: 10.1136/thx.2008.101683 19131451

[13] Ravindran A Ronnberg E Dahlin JS Mazzurana L Safholm J Orre AC. An Optimized Protocol for the Isolation and Functional Analysis of Human Lung Mast Cells. Front Immunol (2018) 9:2193. doi: 10.3389/fimmu.2018.02193 30344519 PMC6183502

[14] Brosseau C Colas L Magnan A Brouard S. CD9 Tetraspanin: A New Pathway for the Regulation of Inflammation? Front Immunol (2018) 9:2316. doi: 10.3389/fimmu.2018.02316 30356731 PMC6189363

[15] Venneker GT Asghar SS. CD59: A Molecule Involved in Antigen Presentation as Well as Downregulation of Membrane Attack Complex. Exp Clin Immunogenet (1992) 9:33–47. 1379443

[16] Qin W Hu L Zhang X Jiang S Li J Zhang Z. The Diverse Function of PD-1/PD-L Pathway Beyond Cancer. Front Immunol (2019) 10:2298. doi: 10.3389/fimmu.2019.02298 31636634 PMC6787287

[17] Huang Z Qi G Miller JS Zheng SG. CD226: An Emerging Role in Immunologic Diseases. Front Cell Dev Biol (2020) 8:564. doi: 10.3389/fcell.2020.00564 32850777 PMC7396508

[18] Bachelet I Munitz A Mankutad D Levi-Schaffer F. Mast Cell Costimulation by CD226/CD112 (DNAM-1/Nectin-2): A Novel Interface in the Allergic Process. J Biol Chem (2006) 281:27190–6. doi: 10.1074/jbc.M602359200 16831868

[19] Andersson CK Tufvesson E Aronsson D Bergqvist A Mori M Bjermer L. Alveolar Mast Cells Shift to an FcepsilonRI-Expressing Phenotype in Mild Atopic Asthma: A Novel Feature in Allergic Asthma Pathology. Allergy (2011) 66:1590–7. doi: 10.1111/j.1398-9995.2011.02723.x 21958156

[20] Sivasubramaniyan K Harichandan A Schumann S Sobiesiak M Lengerke C Maurer A. Prospective Isolation of Mesenchymal Stem Cells From Human Bone Marrow Using Novel Antibodies Directed Against Sushi Domain Containing 2. Stem Cells Dev (2013) 22:1944–54. doi: 10.1089/scd.2012.0584 23406305

[21] Dahlin JS Malinovschi A Ohrvik H Sandelin M Janson C Alving K. Lin- CD34hi CD117int/hi FcepsilonRI+ Cells in Human Blood Constitute a Rare Population of Mast Cell Progenitors. Blood (2016) 127:383–91. doi: 10.1182/blood-2015-06-650648 PMC4731844 26626992

[22] Dahlin JS Ekoff M Grootens J Lof L Amini RM Hagberg H. KIT Signaling is Dispensable for Human Mast Cell Progenitor Development. Blood (2017) 130:1785–94. doi: 10.1182/blood-2017-03-773374 PMC5659818 28790106

[23] Umeda S Kanda M Miwa T Tanaka H Tanaka C Kobayashi D. Expression of Sushi Domain Containing Two Reflects the Malignant Potential of Gastric Cancer. Cancer Med (2018) 7:5194–204. doi: 10.1002/cam4.1793 PMC6198216 30259711

[24] Kiernan JA. Production and Life Span of Cutaneous Mast Cells in Young Rats. J Anat (1979) 128:225–38. PMC1232929 438085

[25] Sperr WR Agis H Czerwenka K Klepetko W Kubista E Boltz-Nitulescu G. Differential Expression of Cell Surface Integrins on Human Mast Cells and Human Basophils. Ann Hematol (1992) 65:10–6. doi: 10.1007/BF01715119 1643154

[26] Agis H Fureder W Bankl HC Kundi M Sperr WR Willheim M. Comparative Immunophenotypic Analysis of Human Mast Cells, Blood Basophils and Monocytes. Immunology (1996) 87:535–43. doi: 10.1046/j.1365-2567.1996.493578.x PMC1384130 8675206

[27] Ghannadan M Baghestanian M Wimazal F Eisenmenger M Latal D Kargul G. Phenotypic Characterization of Human Skin Mast Cells by Combined Staining With Toluidine Blue and CD Antibodies. J Invest Dermatol (1998) 111:689–95. doi: 10.1046/j.1523-1747.1998.00359.x 9764855

[28] Wimazal F Ghannadan M Muller MR End A Willheim M Meidlinger P. Expression of Homing Receptors and Related Molecules on Human Mast Cells and Basophils: A Comparative Analysis Using Multi-Color Flow Cytometry and Toluidine Blue/Immunofluorescence Staining Techniques. Tissue Antigens (1999) 54:499–507. doi: 10.1034/j.1399-0039.1999.540507.x 10599889

[29] Ghannadan M Hauswirth AW Schernthaner GH Muller MR Klepetko W Schatzl G. Detection of Novel CD Antigens on the Surface of Human Mast Cells and Basophils. Int Arch Allergy Immunol (2002) 127:299–307. doi: 10.1159/000057747 12021549

[30] Florian S Sonneck K Czerny M Hennersdorf F Hauswirth AW Buhring HJ. Detection of Novel Leukocyte Differentiation Antigens on Basophils and Mast Cells by HLDA8 Antibodies. Allergy (2006) 61:1054–62. doi: 10.1111/j.1398-9995.2006.01171.x 16918507

[31] Motakis E Guhl S Ishizu Y Itoh M Kawaji H De Hoon M. Redefinition of the Human Mast Cell Transcriptome by Deep-CAGE Sequencing. Blood (2014) 123:e58–67. doi: 10.1182/blood-2013-02-483792 PMC3999759 24671954

[32] Guhl S Neou A Artuc M Zuberbier T Babina M. Skin Mast Cells Develop non-Synchronized Changes in Typical Lineage Characteristics Upon Culture. Exp Dermatol (2014) 23:933–5. doi: 10.1111/exd.12558 25271543

[33] Babina M Guhl S Artuc M Trivedi NN Zuberbier T. Phenotypic Variability in Human Skin Mast Cells. Exp Dermatol (2016) 25:434–9. doi: 10.1111/exd.12924 26706922

[34] Kraft S Kinet JP. New Developments in FcepsilonRI Regulation, Function and Inhibition. Nat Rev Immunol (2007) 7:365–78. doi: 10.1038/nri2072 17438574

[35] Babina M Wang Z Franke K Guhl S Artuc M Zuberbier T. Yin-Yang of IL-33 in Human Skin Mast Cells: Reduced Degranulation, But Augmented Histamine Synthesis Through P38 Activation. J Invest Dermatol (2019) 139(7):1516–25. doi: 10.1016/j.jid.2019.01.013 30684550

[36] Ronnberg E Ghaib A Ceriol C Enoksson M Arock M Safholm J. Divergent Effects of Acute and Prolonged Interleukin 33 Exposure on Mast Cell IgE-Mediated Functions. Front Immunol (2019) 10:1361. doi: 10.3389/fimmu.2019.01361 31275312 PMC6593472

[37] Dwyer DF Ordovas-Montanes J Allon SJ Buchheit KM Vukovic M Derakhshan T. Human Airway Mast Cells Proliferate and Acquire Distinct Inflammation-Driven Phenotypes During Type 2 Inflammation. Sci Immunol (2021) 6(56):eabb7221. doi: 10.1126/sciimmunol.abb7221 33637594 PMC8362933

[38] Oskeritzian CA Zhao W Min HK Xia HZ Pozez A Kiev J. Surface CD88 Functionally Distinguishes the MCTC From the MCT Type of Human Lung Mast Cell. J Allergy Clin Immunol (2005) 115:1162–8. doi: 10.1016/j.jaci.2005.02.022 PMC1460014 15940129

[39] Redegeld FA Yu Y Kumari S Charles N Blank U. Non-IgE Mediated Mast Cell Activation. Immunol Rev (2018) 282:87–113. doi: 10.1111/imr.12629 29431205

[40] Bredenkamp N Stirparo GG Nichols J Smith A Guo G. The Cell-Surface Marker Sushi Containing Domain 2 Facilitates Establishment of Human Naive Pluripotent Stem Cells. Stem Cell Rep (2019) 12:1212–22. doi: 10.1016/j.stemcr.2019.03.014 PMC6565611 31031191

[41] Drew E Merkens H Chelliah S Doyonnas R Mcnagny KM. CD34 is a Specific Marker of Mature Murine Mast Cells. Exp Hematol (2002) 30:1211–8. doi: 10.1016/S0301-472X(02)00890-1 12384153

[42] Drew E Merzaban JS Seo W Ziltener HJ Mcnagny KM. CD34 and CD43 Inhibit Mast Cell Adhesion and are Required for Optimal Mast Cell Reconstitution. Immunity (2005) 22:43–57. doi: 10.1016/j.immuni.2004.11.014 15664158

