# Supplementary material for: Immunoprofiling Reveals Novel Mast Cell Receptors and the Continuous Nature of Human Lung Mast Cell Heterogeneity
Source: Front Immunol. 2022 Jan 4;12:804812. doi: 10.3389/fimmu.2021.804812 (PMC8764255; doi:10.3389/fimmu.2021.804812)
Supplement: Supplementary file 1 [file DataSheet_1.pdf]

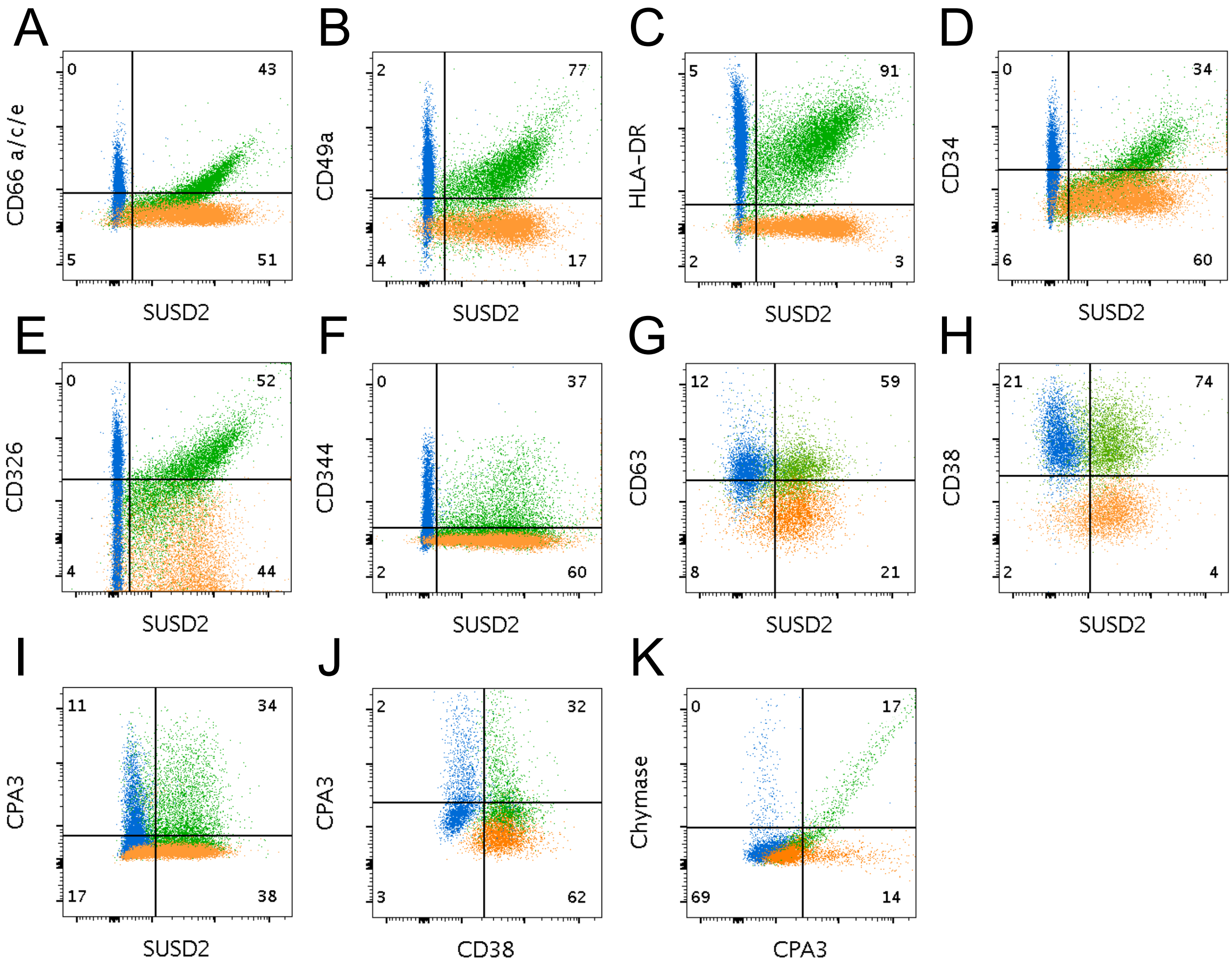

Supplementary Figure S1. Co-stainings including FMO controls

HLMCs were co-stained with indicated markers. Co-stainings are shown in green, respective FMO controls in blue and orange. Isotype controls were used for the intracellular staining of CPA3 and Chymase. Representatives of 3-4 donors are shown.

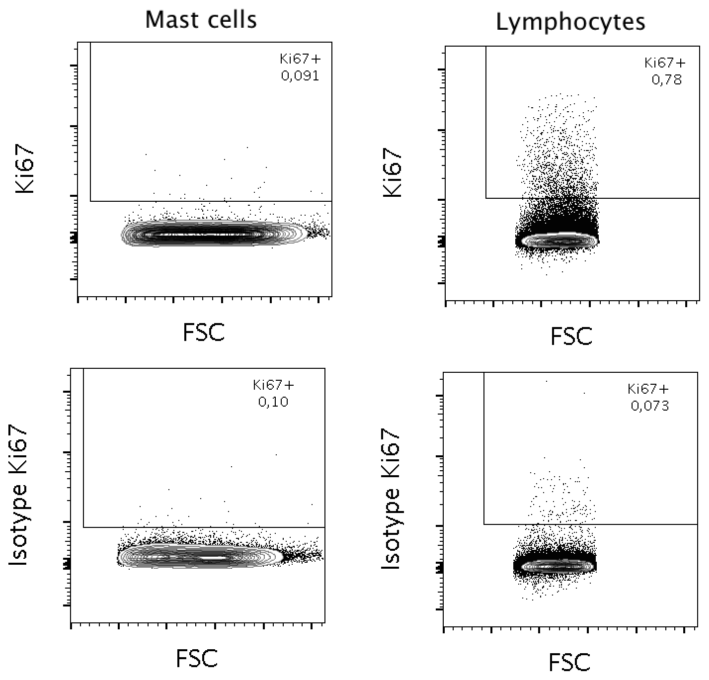

Supplementary Figure S2. No proliferation detected in HLMCs.

HLMCs gated as $CD45^{+}$, $CD14^{low}$ and $CD117^{high}$ were stained intracellularly with the proliferation marker Ki67. As a positive control for the Ki67 staining, cells enriched with lymphocytes were also gated as $SSC^{low}FSC^{low}CD45^{+}$.

Supplementary Table S1. Antibodies included in the LEGENDScreen human cell Screening kit

Specificity	Clone	Specificity	Clone
CD1a	HI149	CD49f	GoH3
CD1b	SN13 (K5- 1B8)	CD50 (ICAM-3)	CBR-IC3/1
CD1c	L161	CD51	NKI-M9
CD1d	51.1	CD51/61	23C6
CD2	RPA-2.10	CD52	HI186
CD3	HIT3a	CD53	HI29
CD4	RPA-T4	CD54	HA58
CD5	UCHT2	CD55	JS11
CD6	BL-CD6	CD56 (NCAM)	HCD56
CD7	CD7-6B7	CD57	HCD57
CD8a	HIT8a	CD58	TS2/9
CD9	HI9a	CD59	p282 (H19)
CD10	HI10a	CD61	VI-PL2
CD11a	HI111	CD62E	HAE-1f
CD11b	ICRF44	CD62L	DREG-56
CD11b (activated)	CBRM1/5	CD62P (P-Selectin)	AK4
CD11c	3.9	CD63	H5C6
CD13	WM15	CD64	10.1
CD14	M5E2	CD66a/c/e	ASL-32
CD15 (SSEA-1)	W6D3	CD66b	G10F5
CD16	3G8	CD69	FN50
CD18	TS1/18	CD70	113-16
CD19	HIB19	CD71	CY1G4
CD20	2H7	CD73	AD2
CD21	Bu32	CD74	LN2
CD22	HIB22	CD79b	CB3-1
CD23	EBVCS-5	CD80	2D10
CD24	ML5	CD81	5A6
CD25	BC96	CD82	ASL-24
CD26	BA5b	CD83	HB15e
CD27	O323	CD84	CD84.1.21
CD28	CD28.2	CD85a (ILT5)	MKT5.1
CD29	TS2/16	CD85d (ILT4)	42D1
CD30	BY88	CD85g (ILT7)	17G10.2
CD31	WM59	CD85h (ILT1)	24
CD32	FUN-2	CD85j (ILT2)	GHI/75
CD33	WM53	CD85k (ILT3)	ZM4.1
CD34	581	CD86	IT2.2
CD35	E11	CD87	VIM5
CD36	5-271	CD88	S5/1
CD38	HIT2	CD89	A59
CD39	A1	CD90 (Thy1)	5E10
CD40	HB14	CD93	VIMD2
CD41	HIP8	CD94	DX22
CD42b	HIP1	CD95	DX2
CD43	CD43-10G7	CD96	NK92.39
CD44	BJ18	CD97	VIM3b
CD45	HI30	CD99	HCD99
CD45RA	HI100	CD100	A8
CD45RB	MEM-55	CD101 (BB27)	BB27
CD45RO	UCHL1	CD102	CBR-IC2/2
CD46	TRA-2-10	CD103	Ber-ACT8
CD47	CC2C6	CD104	58XB4
CD48	BJ40	CD105	43A3
CD49a	TS2/7	CD106	STA
CD49c	ASC-1	CD107a (LAMP-1)	H4A3
CD49d	9F10	CD108	MEM-150
CD49e	NKI-SAM-1	CD109	W7C5

Specificity	Clone
CD111	R1.302
CD112 (Nectin-2)	TX31
CD114	LMM741
CD115	9-4D2-1E4
CD116	4H1
CD117 (c-kit)	104D2
CD119	GIR-208
CD122	TU27
CD123	6H6
CD124	G077F6
CD126 (IL-6Rα)	UV4
CD127 (IL-7Rα)	A019D5
CD129 (IL-9 R)	AH9R7
CD131	1C1
CD132	TUGh4
CD134	Ber-ACT35 (ACT35)
CD135	BV10A4H2
CD137 (4-1BB)	4B4-1
CD137L (4-1BB Ligand)	5F4
CD138	DL-101
CD140a	16A1
CD140b	18A2
CD141	M80
CD143	5-369
CD144	BV9
CD146	SHM-57
CD148	A3
CD150 (SLAM)	A12 (7D4)
CD152	L3D10
CD154	24-31
CD155 (PVR)	SKII.4
CD156c (ADAM10)	SHM14
CD158a/h	HP-MA4
CD158b	DX27
CD158d	mAb 33 (33)
CD158e1	DX9
CD158f	UP-R1
CD161	HP-3G10
CD162	KPL-1
CD163	GHI/61
CD164	67D2
CD165	SN2 (N6- D11)
CD166	3A6
CD167a (DDR1)	51D6
CD169	7-239
CD170 (Siglec-5)	1A5
CD172a (SIRPa)	SE5A5
CD172b (SIRPb)	B4B6
CD172g (SIRPg)	LSB2.20
CD178 (Fas-L)	NOK-1
CD179a	HSL96
CD179b	HSL11
CD180 (RP105)	MHR73-11
CD181 (CXCR1)	8F1/CXCR1
CD182 (CXCR2)	5E8/CXCR2
CD183	G025H7
CD184 (CXCR4)	12G5

Specificity	Clone
CD193 (CCR3)	5E8
CD195 (CCR5)	T21/8
CD196	G034E3
CD197 (CCR7)	G043H7
CD200 (OX2)	OX-104
CD200 R	OX-108
CD201 (EPCR)	RCR-401
CD202b (Tie2/Tek)	33.1 (Ab33)
CD203c (E-NPP3)	NP4D6
CD205 (DEC- 205)	HD30
CD206 (MMR)	15-2
CD207 (Langerin)	10E2
CD209 (DC-SIGN)	9E9A8
CD210 (IL- 10 R)	3F9
CD213a2	SHM38
CD215 (IL- 15Rα)	JM7A4
CD218a (IL-18Rα)	H44
CD220	B6.220
CD221 (IGF-1R)	1H7/CD221
CD226 (DNAM-1)	11A8
CD229 (Ly-9)	HLy-9.1.25
CD231 (TALLA)	SN1a (M3- 3D9)
CD235ab	HIR2
CD243	UIC2
CD244 (2B4)	C1.7
CD245 (p220/240)	DY12
CD252 (OX40L)	11C3.1
CD253 (Trail)	RIK-2
CD254	MIH24
CD255 (TWEAK)	CARL-1
CD257 (BAFF, BLYS)	T7-241
CD258 (LIGHT)	T5-39
CD261	DJR1
CD262	DJR2-4 (7-8)
CD263	DJR3
CD266	ITEM-1
CD267 (TACI)	1A1
CD268 (BAFF-R)	11C1
CD270 (HVEM)	122
CD271	ME20.4
CD273 (B7- DC, PD-L2)	24F.10C12
CD274 (B7- H1, PD-L1)	29E.2A3
CD275 (B7- H2)	9F.8A4
CD276	MIH42
CD277	BT3.1
CD278 (ICOS)	C398.4A
CD279 (PD-1)	EH12.2H7
CD282 (TLR2)	TL2.1
CD284 (TLR4)	HTA125
CD286 (TLR6)	TLR6.127
CD290	3C10C5
CD294	BM16
CD298	LNH-94
CD300e (IREM-2)	UP-H2
CD300F	UP-D2
CD301	H037G3
CD303	201A

Specificity	Clone
CD304	12C2
CD307	509f6
CD307d (FcRL4)	413D12
CD314 (NKG2D)	1D11
CD317	RS38E
CD318 (CDCP1)	CUB1
CD319 (CRACC)	162.1
CD324 (E-Cadherin)	67A4
CD325	8C11
CD326 (Ep- CAM)	9C4
CD328 (Siglec-7)	6-434
CD334 (FGFR4)	4FR6D3
CD335 (NKp46)	9E2
CD336 (NKp44)	P44-8
CD337 (NKp30)	P30-15
CD338 (ABCG2)	5D3
CD340 (erbB2/ HER-2)	24D2
CD344	CH3A4A7
CD351	TX61
CD352 (NTB-A)	NT-7
CD354 (TREM-1)	TREM-26
CD355 (CRTAM)	Cr24.1
CD357 (GITR)	621
CD360 (IL- 21R)	2G1-K12
β2- microglobulin	2M2
BTLA	MIH26
C3AR	hC3aRZ8
C5L2	1D9-M12
CCR10	6588-5
CLEC12A	50C1
CLEC9A	8F9
CX3CR1	2A9-1
CXCR7	8F11-M16
OPRD	DOR7D2A4
DLL1	MHD1-314
DLL4	MHD4-46
DR3 (TRAMP)	JD3
EGFR	AY13
erbB3/HER-3	1B4C3
FcεRIα	AER-37 (CRA-1)
FcRL6	2H3
Galectin-9	9M1-3
GARP (LRRC32)	7B11
HLA-A,B,C	W6/32
HLA-A2	BB7.2
HLA-DQ	HLADQ1
HLA-DR	L243
HLA-E	3D12
HLA-G	87G
IFNGR2	2HUB-159
Ig light chain k	MHK-49
Ig light chain λ	MHL-38
IgD	IA6-2
IgM	MHM-88
IL-28RA	MHLICR2a
Integrin α9β1	Y9A2

Specificity	Clone
integrin β5	AST-3T
integrin β7	FIB504
Jagged 2	MHJ2-523
LAP	TW4-6H10
LT-bR	31G4D8
Mac-2 (Galectin-3)	Gal397
MAIR-II	TX45
MICA/MICB	6D4
MSC (W3D5)	W3D5
MSC (W5C5)	W5C5
MSC (W7C6)	W7C6
MSC and NPC (W4A5)	W4A5
MSCA-1 (MSC, W8B2)	W8B2
NKp80	5D12
Notch 1	MHN1-519
Notch 2	MHN2-25
Notch 3	MHN3-21
Notch 4	MHN4-2
NPC (57D2)	57D2
Podoplanin	NC-08
Pre-BCR	HSL2
PSMA	LNI-17
Siglec-10	5G6
Siglec-8	7C9
Siglec-9	K8
SSEA-1	MC-480
SSEA-3	MC-631
SSEA-4	MC-813-70
SSEA-5	8E11
TCR g/d	B1
TCR Vβ13.2	H132
TCR Vβ23	αHUT7
TCR Vβ8	JR2 (JR.2)
TCR Vβ9	MKB1
TCR Vδ2	B6
TCR Vg9	B3
TCR Vα24- Jα18	6B11
TCR Vα7.2	3C10
TCR α/β	IP26
Tim-1	1D12
Tim-3	F38-2E2
Tim-4	9F4
TLT-2	MIH61
TRA-1-60-R	TRA-1-60-R
TRA-1-81	TRA-1-81
TSLPR (TSLP-R)	1B4
Ms IgG1, κ ITCL	MOPC-21
Ms IgG2a, κ ITCL	MOPC-173
Ms IgG2b, κ ITCL	MPC-11
Ms IgG3, κ ITCL	MG3-35
Ms IgM, κ ITCL	MM-30
Rat IgG1, κ ITCL	RTK2071
Rat IgG2a, κ ITCL	RTK2758
Rat IgG2b, κ ITCL	RTK4530
Rat IgM, κ ITCL	RTK2118
AH IgG, ITCL	HTK888
